# To stay or go? Unpacking the decision-making process and coping strategies of International Medical Graduates practising in rural, remote, and regional Queensland, Australia

**DOI:** 10.1371/journal.pone.0234620

**Published:** 2020-06-16

**Authors:** Bunmi S. Malau-Aduli, Amy M. Smith, Louise Young, Tarun Sen Gupta, Richard Hays

**Affiliations:** College of Medicine and Dentistry, James Cook University, Townsville, Queensland, Australia; Institute of Mental Health, SINGAPORE

## Abstract

Australia is one of many countries to rely on International Medical Graduates (IMGs) to fill general practitioner (GP) positions throughout its regional, rural, and remote (RRR) communities. Current government initiatives requiring IMGs to work for specified periods in RRR areas offer only short-term solutions. The need to improve the long-term retention of IMGs practising in RRR areas has motivated this research to improve our understanding of how IMGs make decisions about where to practise. Specifically, this study sought to: (a) identify the factors that influence an IMG’s decision to remain working in RRR areas, and (b) develop a theory, grounded in the data, to explain how these factors are prioritised, evaluated and used to inform a decision to remain working in RRR areas. This study adopted a qualitative approach and employed grounded theory methods. Data collection and analysis occurred concurrently, using constant, comparative analysis, guided by theoretical sampling and data saturation. Data sources were transcripts from semi-structured interviews with IMG registrars (n = 20) and supervisors (n = 5), interviewers’ notes and analytic memos. Interviewees were all currently working in RRR areas of Queensland, Australia. The analysis involved a three-phase coding process, progressing from specific, inductive coding to abstract, abductive coding. The analysis revealed that the IMG decision-making process involves a complex, dynamic, and iterative process of *balancing life goals based on life stage*. Many factors are considered when assessing the balance of three main life goals: satisfaction with work, family, and lifestyle. The prioritisation and balance of these life goals can vary as the IMG moves through varying work-, family-, and age-related life stages. It is hoped that having this understanding of the complexity of the IMG decision-making process, will better equip medical educators, policy makers and support service providers to tailor services to encourage IMGs to continue practising in these regions.

## Introduction

The recruitment and retention of general practitioners (GPs) to work in regional, rural and remote (RRR) communities remains a challenge for many countries [1]. This includes Australia [2], where maintaining an equitably distributed, sustainable, and well-supported RRR GP workforce has been a long-term problem [3, 4]. Australia is one of many developed countries to rely on International Medical Graduates (IMGs) to fill positions in ‘areas of need’ [2,5–10]. IMGs are doctors who are currently practicing in a country different from the one where they (a) obtained their primary medical qualifications [2,5,8], and (b) were born [4]. They are sometimes referred to as overseas-trained doctors [4,6,7]. IMGs practicing in Australia benefit from government policies that facilitate their immigration to Australia, if they agree to work for a specified period of time in communities with a workforce shortage, known as the ‘ten-year moratorium’ rule [2, 11]. Those who re-train as GPs are required to undertake all GP placements in RRR communities as part of this commitment. As a result, IMGs represent 40% of the Australian RRR GP workforce [6]. However, these are only short-term solutions, as most IMGs relocate to urban areas upon completion of their mandated requirements [12]. Longer-term solutions are more elusive, but could be enhanced by addressing the factors IMGs consider when choosing where to work after their period of compulsory service has ended [2]. Understanding these factors may assist medical educators to tailor educational support services to facilitate IMGs’ decisions to continue practising in RRR areas, where they are needed most.

Prior research has explored factors that influence both GPs [13–16] as a group, and IMGs [7, 8, 17, 18], more specifically, as they decide whether to stay or leave. These decisions are based on the collective assessment of inter-related professional and personal factors [8, 12, 19]. Work satisfaction is based on the scope of practice, work schedules, supporting interactions with management, colleagues and patients, and opportunities for professional development [5, 7, 14, 15, 18, 19]. Personal satisfaction relates to lifestyle preferences, living conditions, and community and family factors [14, 18]. Feeling an attachment, or being part of the local community was important [8, 19]. An ideal community was welcoming, supportive and embracing of differences, with suitable access to facilities and services meeting the needs of the whole family [14, 19]. Religious, ethnic, and cultural considerations involved both community access and acceptance of the IMG’s personal beliefs and customs [7], and their engagement with local people and Indigenous cultures [20]. Family factors included whether community resources met family needs, living arrangements, and time to spend with the family around work demands [7, 12, 14, 16]. Key family-related factors included access to education for children [12, 16] and employment opportunities for spouses/partners [15]. A personal preference for a rural lifestyle was also an important factor [12, 14, 18]. Demographic characteristics influencing a decision included: gender [7, 21], age [16], the location where first settled in Australia, and past rural community experience, including the length of stay at the current location. These factors have been described as both internal (characteristics of the individual) and external (factors relating to the political, economic, and social forces of the community and its geographical location) [14].

Conceptual frameworks were proposed by Hays and colleagues [13] and Humphreys and colleagues [14] to describe how these various factors influence decisions to stay or go for Australia’s rural doctor workforce. The relationship between factors is dynamic, varying across the GP’s lifespan and career stage [13, 14, 16]. Over time, personal and professional needs, life circumstances, and life goals may change, shifting the prioritisation of decision-making factors [14]. The balance between the influences to stay and leave may be disrupted through ‘tipping points’ or ‘triggers,’ such as the oldest child entering secondary school, personality clashes with new colleagues, or poor housing quality [13]. While it is clear that the factors influencing the decision are numerous and diverse, our limited understanding of how these factors are prioritised, weighted, and balanced when making a decision primarily stems from research on GPs practicing in Australia, in general, and not the specific IMG demographic. IMGs, a group that has already made a major, socially dislocating move to a new country, may have a unique decision-making process. Given the importance of IMG retention to the RRR GP workforce, further research is needed to better understand their decision-making process and the role of lifespan and career progression in their decisions. This information can inform educational and community support services to facilitate IMGs’ satisfaction with and decision to remain in RRR practise.

### Study context

In response to the need for GP training in RRR areas, the James Cook University GP Training program (JCU GPTP) was established in 2016 (https://www.jcugp.edu.au/). It is based in North Queensland, Australia, a region with many rural and remote communities distributed over a vast geographic area [22]. JCU’s program adopts a unique distributed model for delivering GP training. GP registrar training is provided within the RRR communities and to the standards of both the Royal Australian College of General Practitioners (RACGP) and Australian College of Rural and Remote Medicine (ACRRM). It is hoped that the RRR-based training will facilitate GPs’ adjustment to the RRR personal and professional lifestyle and remain after training is completed. IMGs make up a substantial proportion of JCU GPTP registrars in regional and rural communities (36%), many of whom are constrained, at least initially, by the ten-year moratorium.

### Research question and aims

This study sought to understand the decision-making context of IMGs training in RRR communities. The specific research question was: How do IMGs practising in RRR Queensland, Australia prioritise their needs and interests when making decisions about whether to continue practising in these areas? There were two aims:

1. Identify the factors that both attract and constrain IMGs’ decisions to go work and remain working in RRR areas.
2. Develop a theory, grounded in the data, to explain how these factors are evaluated, prioritised, and inform the decision-making process.

## Methods

### Study design

This study took an exploratory, interpretivist, and qualitative approach, using grounded theory methods for data collection and analysis. This approach was appropriate because (a) little is known about the substantive area of IMGs deciding to remain working in RRR areas, (b) the aim is to describe a decision-making process, and (c) the outcomes from this study will provide invaluable insights to further improve support services for IMGs working in RRR areas [23–25]. The essential grounded theory methods of concurrent data collection (or generation) and data analysis, theoretical sampling, theoretical saturation, analytic memos, constant comparative analysis that advances through three coding phases, and theoretical sensitivity were used to generate an explanatory theory from interview data [23]. The analysis took a constructivist perspective, whereby the data generated from interviews are believed to have been co-created through the interactions between interviewer and interviewee [26].

### Participant recruitment

The primary source of data for this study was one-on-one, semi-structured interviews conducted either in person or via telephone with IMG Registrars and Supervisors currently working in RRR areas, affiliated with the JCU GP Training Program and listed in the JCU GPTP database. For the purposes of this study, IMGs were defined as GP Registrars and Supervisors who were born and trained outside of Australia, who, at the time of their interview, were living and working in a RRR area of Queensland. To be eligible to participate, the IMG needed to be practising in an area classified as remote, rural or regional, based on the Modified Monash Model (MMM) [27].

### Ethics

Ethics approval (H6771) for this research was granted by James Cook University’s Research Ethics Committee. Participation was confidential and voluntary; consent was obtained either by verbal or written agreement.

### Data collection

Consistent with grounded theory protocols, data collection and data analysis occurred concurrently and were guided by theoretical sampling, where researchers follow leads by sampling new participants or material to provide further clarification on emergent ideas [23–25]. As data were collected and analysed, emergent concepts were identified, requiring additional sampling with modified interview questions to refine and clarify understanding of those concepts.

Data collection occurred in two broad phases from four data sources. Phase 1 involved secondary analysis of data already collected for other purposes. Transcripts or notes of interviews with IMGs practising in RRR areas were purposively sampled from a larger study conducted in 2017, where GPs living and working in rural and remote Queensland were asked questions about their barriers and motivations for living and working in these regions [22]. The sampled transcripts were read and analysed to provide a baseline understanding of the emerging theory and informed subsequent theoretical sampling in Phase 2.

Phase 2 interviews, conducted in 2018, sought to clarify the emerging ideas from the interview transcripts to formulate a theory to explain IMGs’ process for deciding to remain working in RRR areas. Invitations to participate in this round of interviews were emailed to all past IMG study participants (n = 11) and an additional 120 IMG Registrars listed in the JCU GPTP, who were born and trained overseas and working in a RRR area of Queensland. A reminder email was sent one week later. Those who volunteered to participate were entered into a draw to win one of two $100 gift cards. All volunteers were interviewed using a semi-structured interview guide that evolved over time to further refine understanding of emerging concepts. Data were generated through the interaction between interviewer and interviewee. Data collection was terminated when theoretical saturation had been achieved: each of the emerging theory’s key concepts were sufficiently explained; interviews were no longer producing new insights into the theory structure [23, 25]. Interviews were audio recorded and transcribed for data analysis.

Because “all is data” [25, p159] in grounded theory methodology, two other data sources were also incorporated into the analysis process. The analysis considered the interviewer’s detailed notes from the Phase 2 interviews. As part of the concurrent interview data generation and data analysis processes, the researchers recorded their emerging thoughts about the data, codes, categories, and theoretical connections between them throughout the process. These ‘analytic memos’ built a historic audit trail of the emerging and evolving thoughts and linkages proposed by the researchers during the analysis process [23, 24], and were a crucial component of the analysis process.

### Data analysis

Characteristic of grounded theory methods, data were analysed using constant comparative analysis, a systematic process of coding, categorising codes, and developing an explanatory theory that employs both inductive and abductive reasoning [23, 28]. Coding was the analytical process used to identify concepts, compare similarities and differences in the data and to develop a theory that explains the data [23, 24]. NVivo Plus Version 12 (QSR International Pty Ltd., 2018) was used to assist with the data management and organisation of the coding and analysis process.

The constant comparative process undertaken to produce the conceptual framework to explain the IMG decision-making process involved three generalised coding phases, progressing from the specific to more abstract. During initial coding, observations were made through a line-by-line analysis of the interview transcripts to generate initial codes. Incidents were compared with other incidents, looking for similarities and differences, with the goal of conceptualising and categorising the data in terms of beginning patterns in the data. During the intermediate phase of coding, initial codes were compared to other codes, and codes were merged into higher-abstracted categories. The properties and dimensions of the developing categories were refined and the structure or relationship between the categories started to become evident. In this study, the categories fell under the umbrella of a core category, which represented the central phenomenon used to explain the decision-making framework used by IMGs. During advanced coding, the emerging grounded theory was integrated with existing theory to add further explanatory power to the findings. Each phase of coding involved a constant iterative process of identifying consistencies and inconsistences to refine codes, categories, and the relationships between categories to further refine the emerging explanatory theory. This hallmark grounded theory process of constant iterative, dynamic and comparative analysis reflects the combination of inductive and abductive reasoning “that accounts for the conceptual leaps of analysis that occur to move a grounded theory away from being a qualitative descriptive account and towards being an abstract conceptual framework” [23, p91].

### Ensuring methodological rigour

The precise, evidence-based, execution of all recommended grounded theory methods employed in this study, heightened theoretical sensitivity, and helped ensure the methodological rigour of this study, as indicated by the qualitative concepts of credibility, dependability, confirmability and transferability. Additional steps were taken throughout the data collection and analysis process to enhance our sensitivity to identify key data segments. To avoid forming preconceptions that could contaminate the credibility of the emerging theory [25], the researchers refrained from conducting a complete literature review in the substantive area until the final theoretical coding phase of analysis. Self-reflexivity was employed to identify any other potential sources of researcher bias by reflecting on presuppositions. Firstly, we carefully considered the role of each author in the data collection, analysis and interpretation process. While all authors were involved with the conceptualisation and design of this study, BSM-A, LY, TSG, and RH refrained from participating in the interviews and engaging in the initial stages of analysis, due to their prior involvement with IMG recruitment, retention, and education. AMS, an experienced interviewer and qualitative researcher, but who is less familiar with the substantive area, conducted the interviews and initial stages of analysis, discovering the topic and, in consultation with BSM-A, evolving the interview guide as new concepts emerged from the interviews. Throughout all phases of the research, all researchers engaged in self-reflexivity to identify any other potential sources of researcher bias by reflecting on presuppositions. Any potential sources of error were discussed and addressed, to ensure analytical outcomes were appropriately representing interviewee responses.

The study’s dependability (repeatability) and confirmability (objectivity) were targeted through (1) the iterative and concurrent data collection and analysis process and (2) the researchers’ extensive note-taking and memoing, which provided an audit trail for the emerging theory. In grounded theory, the aim is to create a theory with analytic transferability (transferable to expanding/different theoretical propositions) rather than to populations [29]. The structure of the grounded theory process allows for emergent fit: “the theory being used must be constantly compared with the new data, to develop new properties of the categories which show its modified fit, workability and relevance” [25), p.242], meaning that the theory presented here has the potential to be modified to enhance its transferability [25]. By adopting these methods, this research was conducted with meticulous attention to process to ensure quality outcomes with practical relevance to IMGs.

## Findings

### Sample

The data used in this study came from two sources: purposively selected interview transcripts from IMG participants and additional interviews with IMG volunteers. During Phase 1, 11 interview transcripts were obtained and analysed from IMG registrars (n = 6) and supervisors (n = 5). The 17 participants in the Phase 2 interviews included 14 new participant registrars and 3 repeat supervisors from Phase 1. Therefore, the data used in this study were sourced from 25 unique individuals through 28 interviews. At the time of the interviews, all IMGs had placements working in RRR communities in Queensland, Australia (classified as MMM 2–7). IMG participants were working throughout the state, including north, south, east, west, coastal, inland, and island areas. Of the 25 participants, only the five supervisors and 3 registrars were not constrained by the moratorium rule. We spoke with IMGs of both genders and from various stages in their professional and chronological lifespan. All 5 supervisors were male, and 9 registrars were male (11 were female). Supervisors were aged in their 40s (n = 3) and 50+s (n = 2). Registrars were in their 20s (n = 1), 30s (n = 12), 40s (n = 5) and 50s (n = 2). IMG participants came from a variety of family structures. Most participants (n = 23) had a spouse; only 2 IMGs reported being single. Most IMGs had dependent children (n = 20). Of the 16 IMGs who stated their children were school aged, eight were not living with all members of their immediate family.

### Emerging theory: Balancing life goals based on life stage

**Fig 1:**
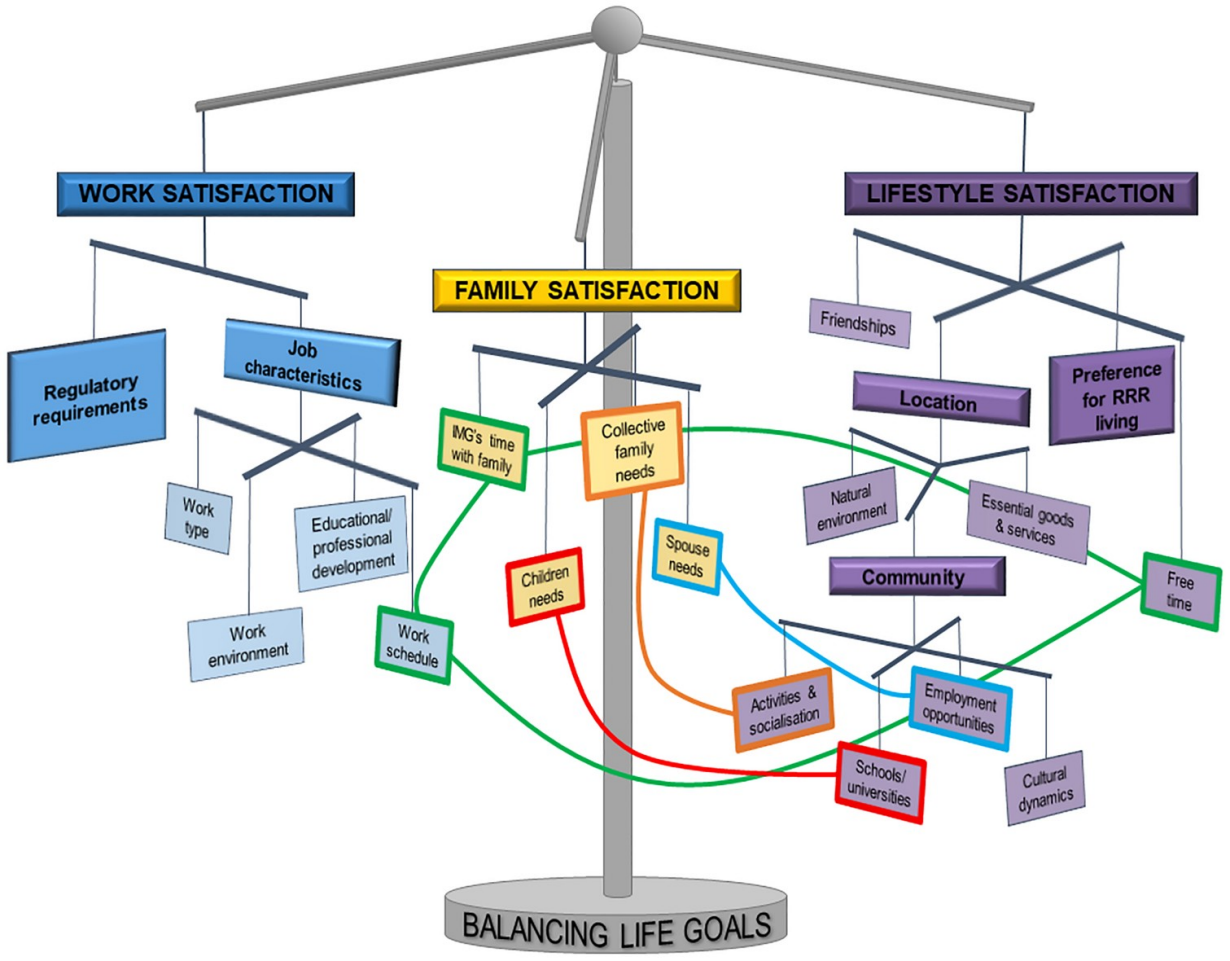
The three inter-related life goals (and their sub-factors) in the *Balancing life goals based on life stage* theory of the IMG decision-making process for choosing to remain working in RRR areas. The coloured lines connecting sub-factors further reflect the inter-relatedness of the life goal balancing act.

This study aimed to explain how IMGs practising in RRR Queensland make decisions about whether to continue practising in these areas. Through grounded theory methods, we first identified the factors used in the decision-making process. Through the increasingly abstracted three-phase coding process, we identified the core category (or central phenomenon) that can explain how these factors influence IMGs’ decision-making process: *balancing life goals based on life stage*. Through a three-pronged prioritisation process, each IMG sought balance in their satisfaction with three primary life goals: work, family and lifestyle (Fig 1). As Supervisor 3 explained in 2017 (S3-17):

… *it’s again looking at their goals*. *I mean*, *they will have professional goals*, *but one must look at their life goals*. *I mean*, *it’s a matter of what do they want out of life*. *Do they want to have a family*? *Do they want to remain single*? *You know*, *as long as their life goals are synchronised*, *as it were*, *with their professional goals*.

While synchronicity may have been the ultimate goal, for many IMGs the decision-making process stopped at finding a balanced level of satisfaction with each life goal based on the current circumstances. How this balance was navigated depended on their life stage and personal preference. *Life stage* includes factors such as the IMG’s career status (e.g., required to work in RRR areas, in training, more senior), family context (e.g., single, married with or without children), and age. As such, the act of balancing these life goals is a dynamic process, one that the IMG will continuously revisit. In the following sections, we will (a) describe how satisfaction with each of the life goals are considered, navigated, and (b) interact with each other within the context of the core category, *Balancing life goals based on life stage*, (c) within the decision-making context of choosing to remain working in RRR areas.

#### Life goal: Work satisfaction

*Work Satisfaction* involved two tiers of consideration, depending on whether the IMG had mandated RRR working requirements. For ‘mandated’ participants, at least temporary satisfaction was achieved by being released from the requirements, at least at a ‘dutiful’ level of satisfaction. However, for all participants, an overall assessment of work satisfaction involved consideration of their satisfaction with several job characteristics: the work type, work schedule, education/professional development opportunities, and the work environment. The *type of work* they were doing that influenced their satisfaction included rural-remote medicine, and preventative and holistic medicine. Others commented on the variety of presentations they saw in a RRR setting, and the continuity of patient care they are able to offer in comparison to urban settings. *Work schedule* considerations were based on workload, flexibility of scheduling, and opportunities for leave. These considerations influenced satisfaction with work-family balance (a Family Satisfaction factor) and work-life balance (a Lifestyle Satisfaction factor). *Educational/ professional development* considerations related to the quality of the work experience they could obtain, and ongoing opportunities to attend conferences and training workshops. *Work environment* considerations focused on evaluations of (1) their relationships with supervisors, co-workers, and patients, and (2) their satisfaction with levels of access to resources (e.g., specialised equipment) to do their job effectively. Work satisfaction was primarily evaluated by the individual IMG and was based on uplifting feelings (of appreciation, fulfilment, being supported, being needed, enjoyment, intrigue, and excitement), and an overall assessment of the suitability of fit between the work and other life goals. A key factor influencing the prioritisation of this life goal is whether the IMG is required to work in RRR areas (rather than being there solely by choice).

#### Life goal: Family satisfaction

An IMG’s decision to remain working in RRR areas is influenced by the satisfaction of the important others in their life. In this case, important others are considered to be those individuals whose needs and best interests are simultaneously considered when making decisions about where the IMG is to work and live. These individuals included partners, children, and extended family, whether living with the IMG or elsewhere. For at least one IMG, the needs of family pets were also important. While many IMGs reported that having friends is an important aspect of their social environment (a Lifestyle Satisfaction life goal), no one indicated that the needs or interests of friends factored into their decision about where to live or work. Therefore, for these IMGs, satisfaction of important others was refined to the family unit. An important distinction between the Family Satisfaction life goal and the Work Satisfaction life goal is in how it is assessed. Work Satisfaction is an individual issue; whereas, Family Satisfaction was collaboratively assessed by the family unit as a whole.

At its core, Family Satisfaction is assessed based on whether the IMG’s living and working arrangements meet the needs of all family members. This was assessed at four different family-dynamic levels based on family satisfaction with meeting the needs and interests of (1) the children (2) the spouse (3) the family unit as a whole, and (4) the IMG (e.g., in terms of satisfaction with the amount of time in physical proximity with the family). The answers to these questions were considered with regards to satisfaction with suitable access to (1) schools and/or universities for children, (2) employment opportunities for spouses, (3) activities suitable for children, spouses, the IMG, and the family as a whole, including sport, theatre, and community events, and (4) other cultural and social support opportunities, including places for religious practice, and opportunities to form friendships and become active members of the community. This means that assessing family-wide satisfaction and suitability with access to activities, social support, education and employment opportunities for the family, involved considerations as to whether the community where the IMG worked offered satisfying opportunities for the family.

For some families, achieving family-wide satisfaction within the context of the IMG’s work commitments meant that family members would spend at least some time throughout the week living apart from each other. A key component to managing the family satisfaction life goal was achieving family-wide satisfaction with the living arrangement configuration. An inter-related aspect to this, is family satisfaction with the IMG’s work-family balance. Is there a satisfactory balance between work and time spent with the family? An important aspect of navigating satisfaction with these alternative living arrangements relates to the IMG’s free time (and availability to the family) and costs and access to suitable means for travel.

The role the *Family Satisfaction* life goal plays in influencing the final decision depends on the family structure and life stage of the family.

#### Life goal: Lifestyle satisfaction

*Lifestyle Satisfaction* relates to the ultimate life goal of living a satisfying life. Lifestyle satisfaction was assessed on two levels of specificity which was based on the IMG’s personal sense of lifestyle satisfaction, and then collectively with the family. Within the context of IMGs making decisions about whether to remain working in RRR areas, lifestyle satisfaction related to four sub-goals.

The sub-goal to live (and work) in an area with a degree of urbanicity (or rurality) that aligns with the IMG’s personal (and family) preference related to the IMG’s (and family’s) feelings of fit with the overall environment. This general preference stems from specific assessments surrounding preferences for the type of lifestyle experienced in RRR areas. It included considerations such as traffic and noise, and general preferences for the RRR laid-back lifestyle. For some IMGs, this general preference formed a defining feature of their identity (e.g., “I’m a country boy,” S1-17).

The sub-goal of achieving free time was another important aspect of the Lifestyle Satisfaction goal. It was assessed based on perceptions of how working and living in this environment would foster personal (and family) health, wellbeing and satisfaction. To meet this goal, considerations were made in relation to whether the work allowed for a satisfactory life outside of work. For some, this related to fostering a preferred workaholic lifestyle; while others sought to ensure they did not work so much that it compromised their personal health and/or their time with the family. This goal is closely linked to the Work Satisfaction life goal of having a satisfying work schedule and the Family Satisfaction life goal of family-wide satisfaction with the IMG’s availability and presence for family time.

Having opportunities and access to friendships was an important consideration for many IMGs as it related to their overall Lifestyle Satisfaction life goal. The desire for meaningful friendships was important to many IMGs, especially when their extended families lived far away (e.g., overseas). Satisfaction with friendships could be achieved by having friendships upon arrival that can be maintained while on the RRR work placement, either via face-to-face or through other satisfactory means of communication (e.g., phone, email, FaceTime). For some, satisfaction with friendships required having opportunities to make friends at the work location. This form of satisfaction is closely linked to another important Lifestyle Satisfaction sub-goal: satisfaction with the location and community.

Satisfaction with location largely involved assessing the suitability of the location in meeting the multi-dimensional needs and interests of the IMG and family unit. Suitability is assessed based on considerations of three broad components:

1. *The natural environment* was assessed based on weather preferences, its perceived beauty, the health of the environment (e.g., air quality), and proximity to desirable natural features (e.g., national parks).
2. *Proximity and access to essential goods and services*. Essential goods that were used in their assessment process included: fuel, clothing, appliances, and access to items specific to their cultural, religious and ethnic heritage (e.g., food). Suitability was assessed based on affordability, convenience, and the time and distance required to travel/ship these items. Proximity to essential services in regional/urban centres, such as transport, were also assessed for suitability based on distance, travel time, travel options, affordability, and convenience.
3. *Community resources and services* were assessed based on their presence, diversity, and quality, and was a central consideration for the Lifestyle Satisfaction life goal. Resources of particular interest to the IMG and the family unit included opportunities for education, employment, extra-curricular activities and socialisation. The community’s cultural dynamics was also an important consideration for IMGs. Consideration is based on access to own cultural and religious practices, the suitability and comfort associated with their fit in the local culture (including Aboriginal culture), and consideration of the inter-cultural relationships between their heritage and local cultures. The overall evaluation of the suitability of the community for the IMG and family unit related to feelings of fit, belonging, and enrichment in terms of their needs being met. Satisfaction on this lifestyle component further informed the Family Satisfaction life goal.

An overall personal (and family-level) preference for RRR living and feelings of fit in the community were important influencing factors in this life goal, both of which were influenced by life stage.

#### Core category: Balancing life goals based on life stage

IMGs frequently reported that these three life goals did not align perfectly, there were some challenges to working in RRR areas, and they had to prioritise and balance their conflicting needs or interests to find a compromise that works for them and their family. How these three life goals were prioritised leading to a decision to remain working in RRR areas depended on where they were at in terms of life stage. Life stage can be conceptualised differently for each life goal.

#### Work priorities

Prioritising work involved two elements of life stage. Work became the penultimate priority for those whose eligibility to work in Australia depended on them working in RRR areas. When an IMG was constrained by the ten-year moratorium or other requirements, then life goals around family and lifestyle took secondary positions. Similarly, an IMG’s life stage in terms of level of GP training also influenced how this life goal is prioritised. Priority was given to work placements that offered opportunities for education/professional development. While this was important to IMGs, regardless of experience level, registrars in the earlier stages of training were willing to make compromises on other life goals to maximise their training opportunities. IMGs found ways to continue working in RRR areas, while making compromises on other life goals to find a, sometimes temporary, balance to the three primary life goals. How IMGs made compromises to other life goals to prioritise work depended on their personal circumstances in terms of overall life stage.

For example, for registrars who are single, without dependents, finding the balance of life goals focuses on satisfaction with lifestyle, work, and important others, which include family who live away (parents, cousins) and friendships. When required to work in a RRR area, the balancing act relates to choosing a RRR work location that will also satisfy lifestyle goals (including goals for friendships or socialisation). For example, one geographically constrained registrar (R8-18) in this category, found balance by choosing a regional location that (a) met her moratorium requirements, (b) had a hospital with all of the sub-specialties she was interested in, (c) where, as she put it, “I pretty much have everything I need,” and (d) where she already had close friends so that she could maintain her social lifestyle of regular catch-ups with friends.

[Choosing this regional centre] *was one of the priorities*, *and because the hospital here had all the sub-specialties*,*… it was* [a] *very appropriate combination of regional and metropolitan*, *so it’s quite urban anyways*, *but I never thought of it as being very remote*.

(R8-18)

When making a decision about whether to continue practising at the particular location (while still constrained by the moratorium), she describes her process as prioritising the nature of the job, and secondarily considering specifics of the location:

*Of course*, *how much rural are we talking for the new job*? *What are the challenges*? *What is the support*? *What is the social support*? *But more importantly*, *what is the professional support*? *If I am in a very difficult situation*, *who can I call for help*, *how far is help*, *how accessible is help*?

(R8-18)

This registrar acknowledges that this prioritisation and balancing of life goals would change if she were to enter into a life stage with a family. She would be even more hesitant to go more rural or remote than her current regional centre: *“and that’s purely for personal reasons*, *because if I had a family … I’d change things”* (R8-18).

A common compromise for an IMG constrained by the moratorium and with a family with school-aged dependent children was for the family to live apart. For example, one strategy to balance children’s education opportunities with IMG RRR living is to send the children to boarding school. An IMG, constrained by the moratorium, with a general preference for practising in RRR areas, and who believed that his children’s education would be compromised if they went to school in his RRR placement, chose to send his 6-, 7-, and 12-year old children to boarding school at the closest regional centre. He and his wife live together. He sees his children on his days off, but when he works weekends, sometimes he doesn’t see his children at all and feels it has impacted upon his relationship with his children. Once he completes his training requirements, he plans to live with his family. While his choice of location was based more on a desire than a requirement to be there, it was the requirement to be there that was compromising his children’s education opportunities and his relationship with them. Once his children finish school, he may choose to return to RRR practice.

*So most of the time I had to live away from my children*, *so it's affected our relationship a little bit—these relationship*, *especially with my older child… Once my children finish their schools*, *I might come back to a regional area to work*. *Yeah*, *because in a way I just want to be close to the family until my children are finished their school education*, *and then*—*for that reason*, *I might move into a regional or urban area*. *Then once they finish*, *I want to move back into a rural setting*, *because I work–like working in regional*—*rural regional areas*.

(R13-18)

Sometimes the IMG lived and worked away while the rest of the family lived in one place. To manage that her family lives away, one registrar said, *“I normally accumulate days*, *and then maybe I can link the four days and then go back and spend time with my family”* (R1-17).

Outside of regulatory requirements and education opportunities, an IMG was likely to prioritise a job placement where they are satisfied with the work environment and type of work they are doing. The level of flexibility in the work schedule was a key factor in being able to find balance with other life goals, and was an important factor in beliefs about having the capacity to stay working in RRR areas.

#### Family commitments

The IMG’s family structure also impacts how these three factors are prioritised. Managing the satisfaction and needs of the family unit vary among life stages, and the RRR areas’ abilities to meet those needs also vary.

For some IMGs, keeping the family together is most important, to help support each other.

*The most important people in my life is just my family*, *my wife and kids*. *They are like shock absorbers for you and sometimes you have ups and downs and stress*, *and sometimes something doesn’t go well you get upset and that is part of work and life*. *So you need some like you need to unwind your stress*, *so you need your partner just to sit and talk and de-stress yourself*.

(R9-18)

An IMG with a partner will incorporate the partner’s needs and desires into any decision to stay. As one Supervisor (with no dependents) explained,

*The thing that unravels everything is the partner*. *If your family's unhappy*, *the whole thing comes unstuck*.

(S5-17)

Compromises for spouse needs included relocating to areas mutually suitable for both IMG and spouse. Some IMGs found satisfaction living on RRR fringes where spouses have access to urban resources and IMGs can commute.

*That is why we decided to live close enough to the border of the regional area so that I could find a job within driving distance from home that would tick those boxes*, *and at the same time my husband still had access to the big city where he works*.

(R11-18)

A lack of employment opportunities or other activities to *“feed”* a partner’s interest can threaten an IMG’s decision to stay. As a Supervisor explains:

*The thing that feed us* [IMGs] *is the work*. *That's a bit selfish because some of our partners are travelling with us and they don’t necessarily get fed*. *They're not all in the medical profession and …* [our current location] *offers her very little*, *because when I come and go she can't work here*, *and she needs to travel with me*.

(S5-17)

For IMGs with children, a decision to continue working in RRR areas also considered the needs, interests, and opportunities for their children, which evolved as they grew up. Families with pre-school-aged children may have their needs met in the RRR community, but concerns about education and access to extra-curricular activities were high-priority decision-making factors for families with school-aged children. For example, a Registrar with two pre-school aged children reported feeling *“pretty happy”* with her current RRR living and working situation, but cited concerns about the quality of the schools as a main consideration for future decisions to remain working in RRR areas.

*So as long as the schools are okay for my kids*, *we would stay*. *But if we have the feeling the schools are not good enough anymore*, *then we will go to a bigger city*.

(R14-18)

Families who prioritised opportunities for their children and found the education and/or extra-curricular activities not suitable in their RRR locations, either relocated or the family lived apart. The aforementioned example of the IMG who lived with his partner, but sent his children to boarding school is one coping strategy some IMGs with primary and secondary school-aged children adopted. Another strategy was for the IMG to live independently while the spouse and children lived elsewhere. These IMGs found creative coping strategies to maintain a satisfactory work-family-life balance.

*This is what I was saying*: *that work-life balance*, *family comes here and I go there sometimes*. *So this is how I'm balancing it*. *At the moment*, *I'm contemplating going part-time next year and see how it works*.

(R1-17)

For one Supervisor, having his first grandchild on the way prompted him to begin to shift his priorities. His wife, who did not have access to all of the activities, resources, employment opportunities, etc., that she desired, was ready to return “home” to live closer to her family, and be closer to their grandchild.

*She's the frustrated party I talk about*, *so yeah…* [she’s] *got to be fed with something*. *Going back to her friends*, *getting to interact with her children and*, *as I said*, *with a grandchild coming… this is an end point*. *If I work in Australia*, *I'll be working on my own*.

(S5-17)

Thus, maintaining family satisfaction is an ongoing process that evolves with one’s ever-changing family circumstances that causes shifts in the prioritisation of preferences to remain working in RRR areas and acts as a key driver in the iterative process of balancing life goals.

#### Lifestyle preferences

For many, the act of balancing work and family needs involved decisions about where and how to live. Lifestyle preferences included considerations about whether the work commitments and location met individual and family needs. Most IMGs reported a preference for RRR living, and a key coping strategy many relied on to find balance was a work schedule that allowed the time needed to find the work-life balance.

One challenge many IMGs described was the additional time and effort required to access essential goods and services while living in RRR areas. IMGs adapted to these issues by working part-time and/or taking leave to make these trips.

Another benefit to working reduced hours was that it allows for more time to enjoy the RRR lifestyle. As one registrar explains, one of the key reasons to stay is the:

… *very nice lifestyle*. *It's not as busy*, *not as fast*, *not as crowded*, *everything is just nice*. *You know*, *you've got short ways everywhere*. *You don’t have to drive so far*. *You get parking spots everywhere*. *You don’t have to pay for everything*. *… The nature is easily accessible*. *The people are usually relaxed and nice*. *Hospitals are small*, *you know*, *more working in a family than like in the big [urban] Hospital*.

(R14-18)

‘Fly in/fly out’ was another strategy that allowed another registrar, constrained by the moratorium, to work RRR while his family remained in the city (and his daughter attended university).

Of all participants in this study, only one overtly stated a dislike for the RRR lifestyle and a preference for urban living.

*Honestly*, *I don’t like living in the rural area*… *If I was given an option to move to … a major city*, *I certain will go*.

(R20-18)

Even this registrar, who was constrained by the moratorium, found a way to prioritise and balance life goals to make a RRR placement work for her circumstances, based on her work-related life stage.

Despite her preference for an urban lifestyle, this registrar still acknowledged a preference for continuing her GP training in a RRR area. She believed that given her junior-level work-related life stage, working in RRR areas offered her more experience (and employment) opportunities.

*If you are beginners*, *for IMGs*, *it’s ideal to start in a rural area*, *because first you get a job opportunity there; second*, *you get more rotations there*, *so you get more experience as well*. … *So you can see like which field are you really interested in*, *like … which one do you want to actually take for future and the third thing is that*, *like obviously*, *it’s easier to understand the health system as well because the population of that area is not as much*. *So*, *the patient turnover is not as much as well*, *so you can take your time to learn stuff and do things*. *… Once you start working there*, *you gain some experience*, *and it’s easier to go to the bigger hospitals* … *So you know the system better*, *and then you are more confident to go to the bigger cities and work there as well*.

(R20-18)

While her work goals were important to her, she is so committed to a lifestyle where her family stays together that she is prepared to stop working to follow her husband, if he took a job in a geographical location where the moratorium would not allow her to work. Her lifestyle satisfaction was closely linked to the abilities of the community’s resources to meet the needs of the family as a whole. At the time of her interview, her husband had about one year left on his moratorium, so they both remained geographically constrained to RRR areas. She reported not liking the feeling that she was mandated to work in a RRR area. She coped with these potentially conflicting objectives of (a) wanting to live in an urban environment, while (b) receiving the extra work-related education and experience that a RRR placement offers, and (c) keeping her family together by living on the urban/regional-rural fringe and commuting daily to a rural area for work. Once her husband enters into the work-related life stage of not being constrained by the moratorium, she is prepared to adjust her priorities and coping strategies. The family-unit will enter into a new life stage and decision-making context–one that allows them to act on their priority preferences for urban living and keeping the family together, even if it means she will cease working. At the essence of this act of balancing life goals was the moratorium mandates. During the moratorium life stage, all other life goals had to accommodate for the mandate.

### Decision-making process

**Fig 2:**
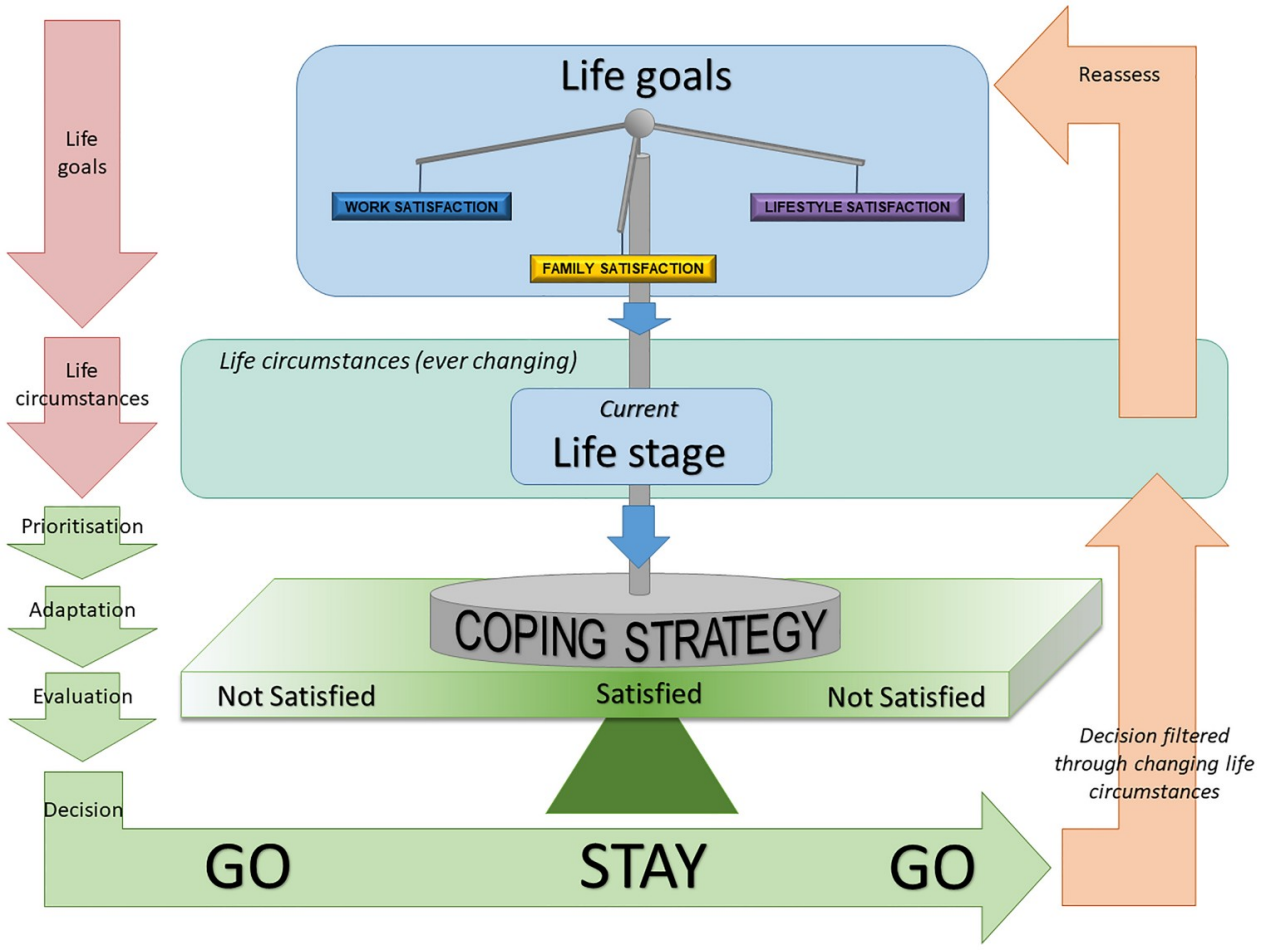
*Balancing life goals based on life stage theory* of the IMG decision-making process for choosing to remain working in RRR areas. The red arrows reflect what each IMG brings to the decision-making context. Green arrows reflect the decision-making process. Orange arrows reflect that this decision-making process is iterative, involving constant reassessment, as life circumstances and life stage change throughout the lifespan.

In summary, we found the IMG decision-making process to be complex, layered and dynamic (Fig 2). IMGs start off with three relatively stable life goals: to maintain satisfaction with work, within the family, and with their lifestyle. Maintaining a satisfactory balance of these three life goals is a complex process involving many factors (Fig 1), and a dynamic process that involves an iterative re-evaluation process as life’s changing circumstances cause IMGs and their families to traverse through different life stages. More specifically, the balancing of life goals are filtered through the IMG’s current life stage. Based on the current needs of the IMG and family, life goals are prioritised, an adaptive coping strategy is implemented. The IMG and family evaluate whether this coping strategy brings satisfactory balance of the life goals, which informs a decision of whether to stay or go. This decision is continuously filtered back through the ever-changing life circumstances and life stage, leading to an ongoing reassessment of *Balancing life goals based on life stage* (Fig 2).

## Discussion

### Summary of key findings

This study sought to generate a theory to explain how IMGs practising in RRR areas prioritise their needs and interests when making decisions about whether to continue practising in these areas. Through a grounded theory analysis, we (a) identified a core category, *Balancing life goals based on life stage*, which involved balancing three life goals, and (b) sought to explain the process. Should the life goals go out of balance, IMGs would assess each goal and prioritise and address the goal(s) upsetting the system’s homeostasis. This prioritisation depended on life stage, and general personal preferences for satisfaction.

### Links to theory

The decision-making framework presented here adds to the growing body of literature seeking to explain the decision-making framework for GPs, and more specifically IMGs, working in Australia’s RRR contexts. Consistent with other studies [8, 12, 14], we found the decision-making factors involved professional considerations and personal lifestyle and family considerations; collectively, the evaluation of these factors involved assessment of social and external (e.g., location) influences. This study offers further support for the importance of maximising IMG workplace retention by fostering a supportive work environment with flexible scheduling, and opportunities for professional development [5, 7, 14, 15, 18]. This study adds to the growing body of international literature that indicates the importance of considering the needs of the family when making decisions about continuing to remain working in RRR areas [12, 16]. Having a preference for RRR living and lifestyle [12, 14, 18] was also supported by the findings from this study. Like other studies [13, 14, 16, 17, 21], we also found there was variation in prioritisation of life goals across the personal and professional lifespan.

Although this study did not set out to empirically test the models developed by Hays et al. [13] and Humphreys et al. [14], our findings support the generalised framework and extends these models to the context of IMGs practising in RRR Australia. At their core, all three models propose that satisfaction with the rural location and a decision to remain working in the RRR area, involve balancing influences to stay and go. Rather than framing these influences in terms of specific factors, as Hays et al. [13] and Humphreys et al. [14] have done, our study views the balancing act around specific life goals (satisfaction at work, with family, and in lifestyle). Our study focusses on how IMGs manage the ‘triggers’ that upset the balance. We offer a description of how IMGs cope, adapt, and manage the dissonance these ‘triggers’ cause, enabling them to choose whether to continue working in RRR areas. We identify that IMGs geographically constrained by the moratorium employ a unique process of prioritisation, whereby they must prioritise their work commitments above all other life goals. This prioritisation and decision-making process was distinct from IMGs not bound by the rules of the moratorium. Although not a specific focus of this study, the absence of literature identifying RRR placements as the penultimate decision-making criteria amongst other GPs practicing in RRR areas would suggest that this is a unique decision-making context for IMGs constrained by the moratorium. We show that while the generalised criteria used to make a decision to remain working in RRR areas largely reflected those criteria identified elsewhere, the prioritisation of these criteria operate differently for IMGs, especially those constrained by the moratorium.

By investigating the perspectives of IMG registrars and supervisors at various stages in their career, family, and age, we were able to further refine prior understandings [14, 19] as to how the prioritisation and balancing of life goals shift across the lifespan, and incorporate this into a model specifically about IMGs. Furthermore, we also add to the Humphreys et al. [14] model the idea that the stress-strain conversion is not solely an individual process. While work factors were individually assessed, family and lifestyle satisfaction were assessed in conjunction with family members, adding to the complexity of the decision-making process. In sum, our *Balancing life goals based on life stage* model, refines our understanding of the decision-making process for IMGs who are constrained and unconstrained as to where they can work by describing the process in terms of balancing life goals across the lifespan.

### IMG uniqueness

While the findings of our study support the generalised framework proposed by Hays et al. [13] and Humphreys et al. [14], it is important to note that the way IMGs navigate this decision-making process is different. IMGs who are constrained by the moratorium, in particular, are known to prioritise their geographical work requirements over all other life goals. Furthermore, it is important to note that the decision-making context for IMGs is different for cultural reasons. IMGs report factors such as access to items specific to their cultural, religious and ethnic heritage (e.g., food) as key determinants of their lifestyle satisfaction. IMGs reported finding work-arounds to obtain satisfaction with these issues, making these issues not penultimate decisions about whether to stay or go. They do, however, act as an example for the ways in which IMGs may have a decision-making context unique from Australian born and trained GPs practicing in RRR areas. The ways in which IMGs adapt to these circumstances they find to be less-than-ideal demonstrates that they are a resilient and adaptive group. While the general criteria and life goals they use to form decisions about whether to stay or go may be reflective of other models broadly describing the decision-making processes of GPs practicing in RRR Australia, the unique decision-making context IMGs find themselves in, suggests that the way they process and prioritise these criteria and life goals may differ from other RRR GPs.

### Coping strategies

IMGs practising in RRR areas have adopted a number of innovative strategies for balancing potentially competing and conflicting life goals to remain working in RRR areas at various work and personal life stages. When balancing life goals, the first priority is requirements. If the IMG is required to work in RRR areas, then compromises will be made to family and lifestyle satisfaction, at least as long as the requirements are in place. For registrars, compromises to family and lifestyle satisfaction are also made, at least temporarily, for the sake of professional education and development opportunities, such as the opportunity to see more diverse presentations and complete rotations in a more timely manner. A general preference for RRR living (and working) is also highly prioritised when making decisions to stay practising in these areas. However, if the family unit is not fully satisfied, this is when a number of adaptive coping strategies are implemented. These strategies often involve the family unit being separated from the IMG’s place of work. This could include: (a) the family living on the fringe of RRR areas and the IMG commuting to work daily, (b) the family living in the city and the IMG practising in a fly-in/fly-out capacity, or (c) the IMG living and working in the RRR area, while the family lives elsewhere and they see each other on holidays or for long weekends.

With regards to access to essential goods, services, activities, and social support, IMGs cope through: online shopping; visits to or residing in more regional/urban centres; and using technology to maintain connections with friends and relatives.

### Recommendations

Through the findings from this study, we were able to identify a number of modifiable (sub) factors influencing decisions, which provide the basis of recommendations for GP training programs and policy and governmental support agencies to help improve IMG retention in RRR areas. As long as the mandated work requirements are in place, staying in an RRR community will be a priority. When the regulatory constraints have been lifted and IMGs are free to make decisions about whether to stay or go, IMG retention may be enhanced by targeting decision-making factors that can be influenced by GP training programs or other IMG support services.

GP training programs’ efforts to support IMGs should consider each situation for the unique circumstances that it brings, and perhaps offer one-on-one consultations/support plans. Each IMG’s effort to balance family and lifestyle satisfaction goals with the work satisfaction goal is an individual effort depending on the unique circumstances of the family, personal preferences for lifestyles and where a person is in terms of their professional and personal lifespan. It is important that GP training programs recognise this when developing support services for IMGs.

IMGs could benefit from a positive work environment with suitable resources to conduct work in a satisfying manner with flexible work schedules. In general, all places of employment should strive for a mutually satisfying work environment and suitable resources to conduct the job. However, the results from this study suggest that an IMG should, in addition, be allowed flexible rostering to balance work and family commitments. This could include rostering that allows for longer breaks with families, while still meeting full-time definitions, similar to fly-in/fly-out (FiFo) miners (who have to travel by air to remote workplaces).

GP training programs and other IMG support programs should offer support not only for the individual IMG, but also for the family, by coordinating with employers and community services. What can the training programs do to further family-orientated activities within the community? Are there ways to form linkages between health care providers and opportunities for school-age children (e.g. distance learning support, ‘summer school’ camps, etc.)? When suggesting placements for IMGs, can employment opportunities for partners be included, perhaps as package deals sponsored by local governments and other employers? Living arrangements should also be part of the employment package. The support needs are likely to be greater where the IMG and family require access to culturally, religiously, and ethnically appropriate opportunities.

Finally, further research is required to better understand, from the IMGs’ perspective, what GP training programs and other IMG support services could do to further assist their continued commitment to work in RRR areas after regulatory constraints are lifted.

#### Strengths and limitations

The findings from this study need to be interpreted within the strengths and limitations of our execution of the essential grounded theory methods. A strength is that we were able to heighten this study’s theoretical sensitivity, credibility, dependability and confirmability. A limitation is that, due to resource constraints, the emerging theory was not presented to participants for feedback and refinement. However, the grounded theory principles of emergent fit are in place, meaning that further refinements and adjustments may be made to the theory as new information emerges. Further, due to time and resource constraints, theoretical sampling was limited. Our study only identified one interviewee who preferred city living; most had a general preference for the RRR lifestyle. Additional purposive sampling for individuals who were unhappy living in the RRR environment could ensure theoretical saturation within the context of the decision-making process of an IMG unhappily working in RRR areas. Finally, the results of this study need to be interpreted with caution when considered outside the context of this study: IMGs, affiliated with JCU’s GP Training program, and practising in RRR Queensland, Australia. It is unknown whether there are differences between volunteer interviewees and IMGs who did not volunteer to participate. Because this study only considered perspectives of IMGs, the comparisons of decision-making criteria between IMGs and other GPs working in RRR contexts were limited to comparisons with the literature. Additional research directly comparing the decision-making processes of domestically-trained GPs and IMGs practicing in RRR contexts would serve to further differentiate the uniqueness of the IMG decision-making process. It is hoped that the findings presented here will inspire other researchers to conduct further research in this substantive area to further expand the analytic transferability of this emerging theory.

### Conclusion

International medical graduates (IMGs) practising in regional, rural and remote (RRR) communities in Queensland, Australia make decisions about whether to remain working in RRR areas by balancing three inter-related life goals: satisfaction with work, family, and lifestyle. A theoretical model is presented to demonstrate how these dynamic factors vary in importance according to life stages and influence decisions about where to practise. Coping strategies may develop when confronting change, resulting in either successful adaptation and a decision to stay, or unsuccessful adaptation and a decision to go. There may be a role for incentives in facilitating IMGs’ decisions to remain working RRR. However, such incentives may need to shift away from the current status quo (financial incentives) toward offering practical assistance to foster retention when pressures to leave increase. Many IMGs commence rural service with established families and more complex support needs and practice requirements by regulatory bodies than domestic graduates. Having an understanding of the IMG decision-making process may better equip medical educators, policy makers and support service providers to tailor services to encourage IMGs to continue to practise in these regions.

## Abbreviations

ACRRM: Australian College of Rural and Remote Medicine
GP: General Practitioner
GT: Grounded Theory
IMG: International medical graduate
JCU GPTP: James Cook University GP Training program
RACGP: Royal Australian College of General Practitioners
RRR: Regional, rural, remote
